# Exploring the operational challenges of navigating ethical oversight in the era of artificial intelligence: a qualitative study of health research ethics committees in Tanzania

**DOI:** 10.1186/s12910-026-01541-0

**Published:** 2026-06-30

**Authors:** Lazaro Amon Solomon Haule, Doreen Mloka, Muhsin Aboud

**Affiliations:** 1 Department of Bioethics and Health Professionalism, School of Public Health and Social Sciences, Muhimbili University of Health and Allied Sciences, P.O. Box 65001, Dar Es Salaam, United Republic of Tanzania; 2 Department of Pharmaceutical Microbiology, School of Pharmacy, Muhimbili University of Health and Allied Sciences, Dar Es Salaam, United Republic of Tanzania; 3 Department of Surgery, School of Medicine, Muhimbili University of Health and Allied Sciences, Dar Es Salaam, United Republic of Tanzania

**Keywords:** Health Research Ethics Committees, Artificial Intelligence, Ethical Oversight, Operational Challenges, Health research governance

## Abstract

**Background:**

Health Research Ethics Committees (HRECs) play a pivotal role in safeguarding research participants and ensuring ethical conduct. The rapid integration of artificial intelligence (AI) into health research introduces novel ethical and operational complexities, including algorithmic opacity, bias, data governance challenges, and difficulties in post-approval monitoring. However, empirical evidence on how these AI-specific complexities affect HREC operations in Tanzania remains limited. This study explored operational challenges associated with ethical oversight of AI-related health research among HRECs in Tanzania.

**Methods:**

An exploratory qualitative study design was employed, involving 25 participants (15 HREC members and 10 secretariat staff) purposively selected from 10 HRECs across five zones of mainland Tanzania. In-depth interviews were conducted using a semi-structured guide. Data were transcribed, translated, and analyzed using inductive content analysis with thematic interpretation, supported by NVivo software. An audit trail, reflexive journaling, and data source triangulation were used to enhance credibility and trustworthiness.

**Results:**

A total of 28 codes were generated and organized into 10 subthemes and four overarching themes. While general challenges such as limited funding, high workload, and staffing constraints persisted, AI-related protocols introduced additional operational burdens, including difficulties in assessing algorithmic validity, increased reliance on external technical experts, and challenges in reviewing large-scale datasets. Although 50% of secretariat staff had more than five years of experience, participants emphasized that the key limitation was not general experience but insufficient AI-specific technical expertise. Weak post-approval monitoring systems were particularly inadequate for tracking AI-driven studies.

**Conclusion:**

Tanzanian HRECs demonstrate foundational governance capacity but face AI-specific operational and technical challenges that constrain effective oversight. Strengthening AI-focused training, technical advisory mechanisms, digital review systems, and sustainable financing is essential to support the ethical governance of emerging technologies.

**Supplementary Information:**

The online version contains supplementary material available at https://doi.org/10.1186/s12910-026-01541-0.

## Introduction

Health Research Ethics Committees (HRECs) play a critical role in safeguarding the rights, welfare, and dignity of human research participants [1, 2]. Their establishment globally followed the atrocities of World War II, when individuals were subjected to lethal medical experiments under the guise of scientific advancement [3]. In response, the Nuremberg Code articulated fundamental ethical principles to prevent exploitation in research [4]. Building on this foundation, international instruments such as the Declaration of Helsinki and CIOMS guidelines provide comprehensive guidance for ethical conduct in human research [5, 6]. While these frameworks set normative standards, HRECs operationalize them by reviewing research protocols to ensure adherence to ethical principles, scientific rigor, and national and international regulations, thereby protecting participants throughout the research process [6, 7].

In many low- and middle-income countries (LMICs), rapid expansion of health research has outpaced the capacity of ethics oversight systems, increasing the risk of ethical lapses, particularly among vulnerable populations [8]. Tanzania reflects this trend, with HRECs formally registered under the National Institute of Medical Research (NIMR) and overseen by the National Health Research Ethics Committee (NatHREC) [9]. Since the first HREC was established in 2002, the number of registered committees has grown to 17 [10, 11], although fewer than ten consistently operate at full capacity. Operational challenges, including limited member expertise, inadequate resources, and weak post-approval monitoring, highlight gaps in research governance and raise concerns about the adequacy, efficiency, and reliability of ethical review in the country’s expanding health research landscape.

The emergence of artificial intelligence (AI) in health research introduces distinct ethical and operational complexities that extend beyond traditional biomedical research oversight [12, 13]. These include challenges related to algorithmic transparency, bias detection, secondary use of large-scale datasets, dynamic consent, and accountability for automated decision-making. Unlike conventional clinical research, AI systems may evolve through continuous learning and data updates, requiring ongoing monitoring, auditing, and ethical oversight rather than a single pre-study ethical approval [14, 15]. These features place additional demands on Health Research Ethics Committees (HRECs), particularly in assessing risks that are not easily observable or predictable.

In Tanzania, ethical oversight of health research is coordinated through the National Health Research Ethics Committee (NatHREC) under the National Institute of Medical Research (NIMR), alongside institutional HRECs [9]. While this system provides a structured framework for ethical review, AI-specific regulatory guidance and technical capacity remain limited. Furthermore, national digital health and data governance frameworks are still evolving, creating uncertainty in the ethical evaluation of AI-driven research.

Despite the increasing adoption of AI in health-related research, including diagnostics, predictive modeling, and digital health systems, there is limited empirical evidence on how HRECs in Tanzania interpret and manage the associated ethical and operational challenges. Existing studies have largely focused on general procedural inefficiencies, such as review timelines and administrative processes, without examining the unique demands posed by AI-related protocols. This study, therefore, addresses this gap by exploring the operational challenges faced by HRECs in navigating ethical oversight in the era of AI, with a focus on identifying context-specific constraints and opportunities for strengthening governance in Tanzania. Insights from this research are critical for strengthening ethical oversight, informing national policy, guiding capacity-building initiatives, and supporting governance frameworks that equip HRECs to safeguard research participants while navigating the ethical complexities of emerging technologies.

## Materials and methods

### Study design and setting

An exploratory qualitative design was employed to investigate the operational challenges faced by Health Research Ethics Committees (HRECs) in the ethical governance of artificial intelligence (AI)-related health research [16]. This design was appropriate because the study aimed to capture participants’ experiences, perceptions, and interpretations of an emerging and underexplored phenomenon. Unlike a case study approach, which focuses on an in-depth examination of a bounded system [17]; this study sought to generate cross-cutting insights across multiple HRECs, making a qualitative exploratory approach more suitable. The study was conducted across 10 registered HRECs located in five zones of mainland Tanzania (Central, Eastern, Lake, Northern, and Southern Highlands). These HRECs operate under the oversight of NIMR and vary in institutional affiliation, operational capacity, and level of functionality. While all are formally registered, some operate at full capacity, whereas others experience resource and staffing limitations. Data were collected between October 2023 and June 2024.

### Sampling and recruitment

Purposive sampling was employed to recruit participants with direct involvement in ethical review processes (HREC members) and those responsible for administrative coordination (secretariat staff), thereby ensuring the inclusion of information-rich participants [18]. In instances where access to participants was constrained, convenience sampling was additionally applied [19]. This combined approach facilitated the inclusion of diverse perspectives across roles, levels of experience, and institutional contexts.

All members and secretariat staff of the selected Health Research Ethics Committees (HRECs) were eligible to participate in the study. An initial target sample of 30 participants was set, guided by the use of both purposive and convenience sampling to enhance methodological rigor [20] Data saturation, defined as the point at which additional interviews no longer yield new insights or contribute to further conceptual development [21], was achieved after 25 interviews, which constituted the final analytic sample. The study was conducted across 10 Health Research Ethics Committees in mainland Tanzania. Through purposive sampling, we recruited both HREC members involved in protocol review and secretariat staff responsible for administrative and coordination functions. This approach enabled the capture of both governance and operational perspectives on ethical oversight, including emerging concerns related to AI (Tables 1 and 2).

**Table 1:** Socio-demographic characteristics of HREC members (*n* = 15)

Socio Biodata Category	Frequency (*n*)	Percentage (%)
Age (Years)		
35–44	7	47%
45–54	5	33%
55–64	2	13%
65–74	1	**7**%
Total	**15**	**100**%
Sex		
Men	8	53%
Women	7	47%
Total	**15**	**100**%
Education		
Bachelor	2	13%
Masters	7	47%
PhD	6	40%
Total	**15**	**100**%
Professional Cadre		
Nurses	1	**7**%
Medical Doctor	5	33%
Public Health Officer	1	**7**%
Veterinary Doctor	1	**7**%
Pharmacist	2	13%
Community Dev. Officer	1	**7**%
Lawyer	3	20%
Information Technologist	1	**7**%
Total	**15**	**100**%
Experience in HREC (Years)		
Under 5	5	33%
Above 5	10	67%
Total	**15**	**100**%
HREC Membership Category		
Chairperson	1	**7**%
Secretary	4	26%
Member	10	67%
Total	**15**	**100**%

**Table 2:** Socio-demographic characteristics of HREC secretariat staff (*n* = 10)

Respondent age in years (Median, (IQR))	41 (38–49)
Age in Years	
35–40	5 (50%)
41–45	2 (20%)
46–53	3 (30%)
Respondent sex	
Female	7 (70%)
Male	3 (30%)
Respondent education	
Diploma	1 (10%)
BSc	2 (20%)
Masters	6 (60%)
PhD	1 (10%)
Years of experience in REC	
1–5	5 (50%)
6–10	3 (30%)
11–15	2 (20%)

### Data collection and analysis process

The semi-structured interview guide was adapted from domains contained in the Research Ethics Committee Assessment Toolkit developed by the African Bioethics Consortium (2017) [22], which is designed to assess operational needs and support quality improvement among research ethics committees. The adapted guide explored participants’ perceptions of the factors and operational challenges influencing HREC functionality, with particular emphasis on ethical oversight in the context of AI-related health research (Supplementary Files 1 and 2). In-depth interviews (IDIs) were conducted by the principal investigator and a trained research assistant in either Swahili or English, according to participants’ language preferences. This approach facilitated open communication and enabled participants to express their views accurately and comfortably. The interview guide was originally developed in English, translated into Swahili, and reviewed by bilingual experts to ensure conceptual equivalence between the two versions. Interviews lasted between 30 and 60 min and were audio-recorded with participants’ consent. All interviews were conducted in private settings to ensure confidentiality, and written informed consent was obtained from every participant. Anonymous identifiers were assigned to protect participants’ identities.

All interviews were transcribed verbatim. Interviews conducted in Swahili were translated into English by experienced bilingual personnel. An independent language expert reviewed both the original transcripts and translated versions to verify accuracy and ensure that participants’ intended meanings were preserved. Data were analyzed using inductive content analysis, involving open coding, grouping of codes into categories, and abstraction into broader themes [23, 24], thereby enabling findings to emerge directly from participants’ accounts rather than being guided by predefined frameworks. The analysis proceeded in three stages: (1) open coding, (2) grouping codes into subthemes, and (3) synthesis into overarching themes (Tables 3 and 4).

**Table 3:** Codes, subthemes, and overarching themes (*N* = 15 HREC Members)

S/*N*	Codes	Subthemes	Overarching Theme
1	Availability of policies; Availability of Standard Operating Procedures (SOPs); Availability of guidelines	Adequacy of ethics governance instruments as perceived by REC members	Administrative and Governance Factors Shaping REC Functionality
	Capacity for review; REC capability to review proposals	Perceived technical and procedural competence of RECs	
	Autonomy in decision-making; Semi-autonomy in decision-making; Signatories belonging to a higher authority	Institutional independence and limits to REC decision-making authority	
	Diversity of REC membership and expertise; Adequacy of REC membership; Membership turnover	Composition, expertise diversity, and continuity of REC membership	
2	Insufficient funding; Budget allocation for the REC; Funding from researchers’ contributions; Reliance on submission fees	Financial constraints affecting REC operations	Capacity Constraints and Operational Challenges
	Lengthy waiting periods; Complaints from principal investigators (PIs)	Delays in ethical review and perceived accountability pressures	
	High workload; High volume of protocol submissions; Limited number of reviewers; Reviewers’ competing institutional responsibilities	Workload pressure and human resource limitations	
	Availability of material resources; Support staff availability; Compensation for time and effort	Inadequate administrative and logistical support	
	Access to relevant expertise; Capacity-building initiatives	Gaps in specialized expertise and training opportunities	
	Follow-up of approved protocols; Oversight remains a challenge for most RECs	Weak post-approval monitoring and oversight practices	

**Table 4:** Codes, subthemes, and overarching themes (*N* = 10 heads of HREC secretariats)

Overarching Themes	Subthemes	Codes	Illustrative Quote (Heads of HREC Secretariats)
1. Administrative Structures and SOPs	SOPs Not AI-Specific	SOP limitations for AI review	“Our SOPs cover general research review, but do not specifically address AI protocols. We often have to interpret existing guidelines, which slows down the review process.” – Head of HREC Secretariat #1
	Governance and Independence	Operational autonomy; limited AI guidance	“We operate independently, which allows us to enforce deadlines and ensure compliance. However, AI studies are complex, and current checklists provide limited guidance.” – Head of HREC Secretariat #4
	Documentation Challenges	Inadequate tracking systems	“Tracking AI protocols is difficult because our database was not designed for complex, data-driven studies. Updates are often delayed, and files can be misplaced.” – Head of HREC Secretariat #7
2. Operational and Resource Constraints	High Workload	Administrative burden	“Even routine protocol management demands significant time. AI studies require additional coordination, extra meetings, and frequent follow-ups with reviewers.” – Head of HREC Secretariat #2
	Staff Shortages	Limited personnel	“We have limited administrative staff. Processing AI-driven protocols often leads to backlogs, especially when external reviewers are unavailable.” – Head of HREC Secretariat #5
	Limited IT and Analytical Tools	Insufficient infrastructure	“We lack systems to validate AI datasets or monitor algorithmic outputs, making administrative oversight more challenging.” – Head of HREC Secretariat #8
3. Training and Capacity-Building Needs	Lack of AI-Specific Training	Training gaps	“Most of our training focuses on conventional ethics and clinical trials. There is minimal guidance on AI research, leaving us unsure how to support the committee effectively.” – Head of HREC Secretariat #3
	Need for External Expertise	Dependence on technical advisors	“We frequently rely on external reviewers or technical advisors. Coordinating them adds extra administrative workload.” – Head of HREC Secretariat #6
4. Monitoring and Compliance Challenges	Follow-Up Difficulties	Post-approval monitoring gaps	“Post-approval monitoring of AI research is nearly impossible with current resources. We cannot systematically track how algorithms impact participants.” – Head of HREC Secretariat #9
	Ethical Oversight Uncertainty	Difficulty assessing algorithmic risk	“Administratively, we can ensure that forms are complete, but assessing algorithmic fairness or bias is beyond our current capacity.” – Head of HREC Secretariat #10

An audit trail documenting coding decisions, theme development, and analytical reflections was maintained throughout the process. Coding was iterative and involved regular discussions among the research team to enhance credibility and consistency. Although content analysis was the primary approach, thematic interpretation was used to deepen understanding of patterns and relationships within the data [25], particularly in relation to AI-related ethical challenges, enabling a more interpretive analysis of underlying meanings and connections across themes (Table 5).

**Table 5:** Converged matrix of codes, subthemes, and overarching themes from HREC Members (*n* = 15) and Heads of HREC Secretariat (*n* = 10)

Overarching Theme	Subtheme	Codes from HREC Members	Codes from Heads of HREC Secretariats
1. Administrative and Governance Structures Shaping Ethical Oversight	Adequacy of policies, SOPs, and guidelines	Availability of policies; Availability of SOPs; Availability of guidelines; Capacity for protocol review	SOPs not AI-specific; SOP limitations for AI review
	Governance and institutional independence	Autonomy in decision-making; Semi-autonomy in decision-making; Signatories belonging to higher authority	Operational autonomy; Limited AI governance guidance
	Committee composition and expertise	Diversity of HREC membership; Adequacy of membership; Membership turnover	Dependence on external technical advisors
	Documentation and information systems	HREC capability to manage review processes	Inadequate tracking systems; Documentation challenges
2. Operational and Resource Constraints Affecting HREC Performance	Financial and material resource limitations	Insufficient funding; Reliance on submission fees; Budget constraints	Limited IT and analytical tools; Insufficient infrastructure
	Human resource shortages	Limited number of reviewers; Competing responsibilities	Staff shortages; Limited administrative personnel
	Workload and review pressure	High workload; High volume of protocol submissions	Administrative burden; Increased coordination demands for AI studies
3. Capacity, Training, and Expertise Gaps in Emerging Research Areas	Limited AI-specific knowledge and skills	Limited access to specialized expertise; Capacity-building gaps	Lack of AI-specific training; Training gaps
	Dependence on external expertise	Need for external reviewers	Reliance on technical advisors; Coordination challenges
4. Monitoring, Compliance, and Post-Approval Oversight Challenges	Weak post-approval monitoring	Follow-up on approved protocols; Oversight remains a challenge	Post-approval monitoring gaps; Follow-up difficulties
	Ethical risk assessment limitations	Difficulty assessing complex protocols	Difficulty assessing algorithmic risk; Ethical oversight uncertainty

### Research team and reflexivity

The research team comprised the principal investigator, trained in bioethics and qualitative research, and a research assistant with prior experience conducting semi-structured interviews. Both researchers received additional training in qualitative interviewing, research ethics, confidentiality, and systematic reflexive qualitative inquiry before data collection. To ensure reflexivity and minimize researcher bias, reflexive journaling was maintained throughout all stages of the study, including data collection, transcription review, and analysis. The researchers documented their assumptions, methodological decisions, emerging interpretations, and reflections on participant interactions [26, 27]. These reflexive notes were discussed in regular analytic debriefing sessions and used as an integral part of the coding and interpretation process to critically interrogate how the researchers’ disciplinary backgrounds, assumptions, and positionality could shape meaning-making. This iterative reflexive process enhanced analytical transparency, reduced interpretive bias, and strengthened the credibility and dependability of the findings [28].

### Trustworthiness and rigor

Trustworthiness was ensured in accordance with Lincoln and Guba’s criteria for qualitative rigor, credibility, dependability, confirmability, and transferability [29, 30]. Multiple strategies were applied across data collection, analysis, and interpretation to ensure methodological robustness. Credibility was strengthened through triangulation of data sources, including in-depth interview transcripts, field notes, and iterative coding conducted collaboratively by the research team. In addition, ongoing participant clarification (member checking during interviews) was used to verify interpretations and ensure accurate representation of participants’ views. Dependability was ensured through the maintenance of a comprehensive audit trail documenting all stages of the research process, including raw data management, coding procedures, theme development, and analytic decisions. This audit trail provided a transparent and verifiable account of the analytic process, enabling assessment of methodological consistency. Confirmability was enhanced through systematic reflexive journaling and independent peer review of emerging codes and themes within the research team, which minimized individual bias and ensured that findings were firmly grounded in participant narratives rather than researcher assumptions. Transferability was supported through a thick description of the study setting, institutional contexts, participant characteristics, and HREC operational environments. This level of contextual detail enables readers to assess the applicability of findings to similar ethical review systems in other low- and middle-income country settings.

### Data management and security

All data, including audio files, transcripts, and translated documents, were stored in encrypted, password-protected digital folders accessible only to the research team. Hard-copy materials were kept in locked storage. Identifiers were removed during transcription, and pseudonyms were used throughout analysis to maintain participant anonymity. Data handling complied with MUHAS and NIMR data protection regulations. All data will be retained for five years post-publication and subsequently destroyed securely.

### Ethics approval and consent to participate

This study adhered to the principles outlined in the Declaration of Helsinki and relevant international ethics guidelines for research involving human participants [5, 9, 31]. Ethical approval was obtained from the Institutional Review Board (IRB) of Muhimbili University of Health and Allied Sciences (MUHAS) (Ref. No. DA 282/298/01 C/1010, approved March 9, 2022) and the National Health Research Ethics Committee (NatHREC) of the National Institute of Medical Research (NIMR) (Ref. No. NIMR/HQ/R.8a/Vol.IX/4017, approved June 7, 2022).

Administrative clearance was secured from the MUHAS Directorate of Postgraduate Studies, and the heads of the relevant research and academic institutions granted formal permission. Participants received detailed information regarding the study’s objectives, procedures, potential risks, and anticipated benefits. Participation was voluntary, and all participants provided written informed consent. They were informed of their right to withdraw at any point without penalty. Confidentiality and anonymity were ensured through de-identification and secure data storage. The study design upheld the ethical principles of respect for autonomy, beneficence, non-maleficence, and justice, in line with internationally recognized bioethical standards [32].

### Reporting standards

This study followed the Consolidated Criteria for Reporting Qualitative Research (COREQ) 32-item checklist [33, 34], which guides transparent reporting of qualitative research using interviews. Adherence to COREQ ensured comprehensive documentation of the research team, study design, data collection, analysis, and ethical considerations.

## Findings

### Participant characteristics

This study included 25 purposively selected participants drawn from 10 Health Research Ethics Committees (HRECs) in mainland Tanzania, comprising 15 HREC members and 10 Heads of HREC Secretariats. Participants were predominantly middle-aged, with most aged 35–54 years; secretariat respondents had a median age of 41 years (IQR: 38–49). Sex distribution varied by role, with men slightly predominating among HREC members (53%), while women comprised the majority of heads of HREC secretariat (70%). Educational attainment was high, with 80% of participants holding postgraduate qualifications. The sample reflected multidisciplinary representation, including medicine, public health, pharmacy, nursing, veterinary medicine, law, bioethics, administration, information technology, and community development. Experience in ethical review varied: 67% of HREC members had more than five years of service, whereas 50% of secretariat respondents had served for 1–5 years, providing complementary governance and operational perspectives on HREC functioning. Detailed socio-demographic characteristics of HREC members and secretariat respondents are presented in Tables 1 and 2, respectively.

### Overview of key findings

Interviews with HREC members and heads of HREC secretariat generated 28 codes, organized into 10 subthemes and 4 overarching themes (Table 5). The subthemes included: adequacy of policies and SOPs, governance and independence, committee composition and expertise, documentation and information systems, resource and human capacity, workload, training and expertise gaps, reliance on external expertise, post-approval monitoring, and oversight mechanisms. These were grouped under the overarching themes: (1) Administrative and Governance Structures Shaping Ethical Oversight, (2) Operational and Resource Constraints Affecting HREC Performance, (3) Capacity and Training Gaps in Emerging Ethical Oversight Challenges, (4) Monitoring, Compliance, and Post-Approval Oversight Challenges. These themes reflect the perceived factors and operational challenges influencing ethical oversight in the era of AI health-related studies, integrating governance perspectives from HREC members and practical experiences from secretariat staff.

### Theme 1: administrative and governance structures shaping ethical oversight

Findings indicate that administrative and governance structures are central to the effective functioning of Health Research Ethics Committees (HRECs). Participants emphasized that formal policies, standard operating procedures (SOPs), committee composition, and operational independence collectively support consistent, transparent, and accountable ethical review processes. However, the emergence of AI-related health research introduces novel ethical and technical challenges that are not fully accommodated within existing governance frameworks.

#### Adequacy of policies and standard operating procedures (SOPs)

Participants reported that clear policies and SOPs are essential for guiding the submission, review, and approval of research protocols. These frameworks were described as important for standardizing procedures, reducing ambiguity, and ensuring consistency in decision-making. However, most committees rely on national-level guidelines, with limited contextual adaptation to emerging areas such as AI-related health research. As a result, some ethical issues associated with AI studies are not explicitly addressed, requiring committees to rely on interpretation and professional judgment during review.

*“We have written documents and research policies in place. They are well-documented and correctly formulated. They guide us on how to manage submissions*,* reviews*,* and approvals systematically*,* which reduces conflicts during deliberations.” {HREC member #01}*.

*“We don’t have our own policies; we use those developed by the National Institute for Medical Research. While this ensures alignment with national standards*,* sometimes locally relevant ethical issues*,* such as those arising in AI health-related studies*,* are not fully addressed*,* and we have to improvise.” {HREC member #02}*.

Secretariat staff further noted that general SOPs are often insufficient for guiding the review of AI-related protocols:

*“Our SOPs cover general research review but do not specifically address AI health-related studies. We often have to interpret existing guidelines*,* which slows down the review process*,* especially when we encounter studies with complex algorithms or large health datasets.” {Head of HREC Secretariat #1}*.

Overall, these findings suggest that while existing governance frameworks provide essential procedural structure, they are not sufficiently responsive to the evolving demands of AI-related health research. The reliance on generalized SOPs necessitates interpretive flexibility, which may introduce variability in review processes and underscores the need for more adaptive and technology-sensitive regulatory guidance.

#### Committee composition and governance

Participants highlighted that multidisciplinary committee composition enhances ethical deliberation by incorporating diverse professional, scientific, and social perspectives. This diversity was considered essential for a comprehensive evaluation of both conventional and AI-related health research.

*“Our committee is diverse*,* including priests*,* social workers*,* scientists*,* pharmacists*,* nurses*,* and doctors. This diversity strengthens our decision-making process*,* allowing us to consider scientific*,* ethical*,* and social implications comprehensively*,* including those arising in AI health-related studies.” {HREC members #08}*.

Operational independence was also described as a key factor supporting effective and unbiased decision-making:

*“Our committee functions independently without external interference. We make decisions collectively*,* and once a decision is made*,* it remains final. This autonomy is crucial to uphold ethical standards*,* even when institutional pressures exist.” {HREC members #15}*.

However, some participants reported occasional external interference in approval processes, which may affect efficiency and timeliness.

*“There is a challenge in our approval process. Typically*,* the chairperson and secretary should sign off on approvals*,* but higher authorities sometimes intervene*,* causing unnecessary delays. This undermines our efficiency and credibility*,* especially for AI health-related studies that require careful deliberation.” {HREC members #09}*.

These findings suggest that while multidisciplinary composition and autonomy strengthen ethical governance, institutional interference can compromise procedural efficiency. This reflects a structural tension between independence and hierarchical oversight, which becomes more pronounced when dealing with complex and time-sensitive AI-related protocols.

#### Documentation and tracking systems

Effective documentation and tracking systems were described as essential for ensuring transparency, accountability, and continuity in ethical oversight. However, participants noted limitations in existing systems, particularly in managing complex and data-intensive AI-related studies.

*“Tracking AI health-related studies is difficult because our database was not designed for complex*,* data-driven studies. Updates are often delayed*,* and files can be misplaced*,* which complicates oversight.” {Head of HREC Secretariat #7}*.

This finding indicates that existing documentation systems are not adequately designed to accommodate the complexity of AI-driven research. Weak digital infrastructure limits traceability and timely monitoring, thereby constraining effective oversight and accountability in evolving research environments.

### Theme 2: operational and resource constraints affecting HREC performance

Findings indicate that operational and resource constraints significantly affect the efficiency of HREC functioning. These constraints impact all stages of ethical review, including protocol assessment, administrative coordination, and monitoring activities. The challenges are further amplified by the technical complexity of AI-related health research.

#### Limited funding and resources

Participants consistently identified inadequate funding as a major barrier to effective HREC operations. Limited financial resources affect committee meetings, reviewer engagement, and timely protocol review.

*“Limited funding affects our ability to deliver optimal services. Without sufficient funds*,* some protocols remain unreviewed for two to three months*,* leading to complaints from principal investigators.” – HREC members #05*.

*“Committee meetings require member stipends*,* and without funds*,* we cannot convene regularly. This hampers timely reviews and oversight*,* particularly for AI health-related studies that require specialized evaluation.” {HREC members #03}*.

Secretariat staff further highlighted infrastructural limitations, especially the absence of systems capable of supporting AI-related data validation and oversight.

*“We lack systems to validate AI health-related datasets or monitor algorithmic outputs*,* making administrative oversight more challenging. This affects our ability to ensure ethical compliance.” {Head of HREC Secretariat #8}*.

These findings suggest that financial and infrastructural constraints significantly weaken both procedural efficiency and technical oversight capacity. In particular, the lack of dedicated resources for AI-related evaluation limits the committees’ ability to respond effectively to emerging ethical complexities.

#### Workload and staffing limitations

High workload and insufficient staffing were reported as major barriers to timely and effective ethical review. Participants noted that AI-related studies require additional coordination and technical input, further increasing administrative burden.

*“Even routine protocol management is time-consuming. AI health-related studies require extra coordination*,* additional meetings*,* and frequent follow-ups with reviewers*,* stretching our capacity.” {Head of HREC Secretariat #2}*.

*“Processing AI health-related studies often results in backlogs*,* especially when external reviewers are unavailable. Administrators have to balance other institutional duties alongside these responsibilities.” {Head of HREC Secretariat #5}*.

These indicate that existing human resource structures are overstretched and not adequately aligned with the demands of increasingly complex research protocols. The added burden of AI-related studies exacerbates delays and reduces overall efficiency in ethical review processes.

### Theme 3: capacity and training gaps in emerging ethical oversight challenges

The findings reveal significant gaps in technical capacity among HREC members and secretariat staff, particularly in relation to AI-related health research. These gaps limit the ability to effectively assess algorithmic systems and emerging ethical risks.

#### Lack of AI health-related training

Participants reported limited exposure to AI-focused ethical training, with most capacity-building initiatives concentrating on conventional clinical research ethics.

*“Most of our training focuses on conventional ethics and clinical trials. There is minimal guidance on AI health-related studies*,* leaving us uncertain how to support the committee effectively.” {Head of HREC Secretariat #3}*.

This finding suggests a mismatch between existing training frameworks and emerging technological demands. The lack of AI-specific capacity building limits preparedness and reduces the ability of committee members to critically engage with algorithmic and data-driven research ethics.

#### Reliance on external expertise

Due to limited internal expertise, committees frequently rely on external reviewers or technical experts, which introduces coordination challenges and delays.

*“We frequently rely on external reviewers or technical advisors. Coordinating them adds extra administrative workload*,* and scheduling delays can affect protocol timelines.” {Head of HREC Secretariat #6}*.

This finding indicates structural dependency on external expertise due to internal capacity gaps. While this improves technical rigor, it also introduces inefficiencies and delays, highlighting the need for strengthened internal capacity to ensure sustainable ethical oversight.

### Theme 4: monitoring, compliance, and post-approval oversight challenges

Findings show that post-approval monitoring remains a weak component of HREC functioning due to resource and capacity limitations. This challenge is particularly evident in the oversight of AI-related health research.

#### Post-approval monitoring

*“With current resources*,* we cannot systematically track how AI health-related protocols impact participants. Post-approval monitoring is nearly impossible given staff and financial limitations.” {Head of HREC Secretariat #9}*.

This finding suggests that post-approval oversight systems are underdeveloped, limiting the ability of HRECs to ensure ongoing compliance and participant protection. The dynamic nature of AI systems further intensifies this gap, as continuous monitoring is required but not feasible under current constraints.

#### Ethical oversight limitations

*“Administratively*,* we can ensure forms are complete*,* but evaluating algorithmic fairness or bias is beyond our capacity. This leaves a gap in identifying potential ethical risks in AI health-related studies.” {Head of HREC Secretariat #10}*.

The finding indicates a clear divide between administrative compliance and substantive ethical evaluation. While procedural requirements are met, deeper algorithmic risks remain insufficiently assessed, revealing a significant governance gap in AI-related ethical oversight.

## Discussion

This study examined the operational challenges faced by Health Research Ethics Committees (HRECs) in overseeing artificial intelligence (AI)-related health research. The findings indicate that while many challenges reflect long-standing structural constraints within low- and middle-income countries (LMICs), AI introduces additional governance demands that significantly reshape ethical review processes. These include algorithmic complexity, data governance challenges, and the need for continuous oversight mechanisms. At a global level, similar concerns have been raised regarding the adequacy of traditional ethics review systems in addressing AI in health research, with increasing calls for adaptive and lifecycle-based governance approaches rather than static approval models [35].

A key finding of this study is that AI-related health research imposes fundamentally different ethical review requirements than conventional clinical research. Unlike static interventions, AI systems evolve through training, retraining, and continuous data updates, making their risk profiles dynamic rather than fixed. This finding aligns with a growing body of global literature emphasizing that artificial intelligence (AI) systems introduce ongoing and dynamic risks, such as model drift, algorithmic opacity, and embedded bias, that cannot be fully anticipated or assessed at the point of initial ethical approval [36]. In the context of Tanzania, these concerns are particularly salient given the country’s rapid adoption of AI in the health sector alongside evolving regulatory and governance frameworks. Evidence indicates that existing procurement, policy, and ethics oversight mechanisms in Tanzania are still largely designed for conventional technologies and struggle to adequately address the complexity, adaptiveness, and context-sensitivity of AI systems.

Furthermore, broader African scholarship highlights that many prevailing AI ethics frameworks are externally derived and may inadequately capture local realities, thereby increasing the risk of bias, inequity, and unintended harms if systems are not continuously monitored and contextually adapted [37]. In line with our findings, this underscores the need for shifting from static, one-time ethical approval models toward adaptive, lifecycle-based governance approaches that incorporate ongoing evaluation, local contextualization, and accountability mechanisms throughout the deployment of AI in health research and practice. Evidence from East Africa similarly indicates that research ethics systems remain largely oriented toward conventional biomedical research, with limited preparedness for governing algorithm-driven health technologies. Studies across the region highlight gaps in regulatory capacity, digital health governance, and ethical oversight frameworks, which are often not adequately equipped to address the unique challenges posed by AI, including data governance, accountability, and continuous system evolution [16, 38]. This reinforces the relevance of the current findings, which demonstrate that HRECs are required to interpret AI protocols without sufficient technical scaffolding.

The study found that existing SOPs and national ethical guidelines provide an essential procedural foundation but remain insufficient for addressing AI-specific ethical challenges, including algorithmic transparency, bias detection, and dataset validation. This aligns with global literature, which highlights that AI governance frameworks are often reactive rather than anticipatory, struggling to keep pace with rapid technological advancement [39]. While some high-income countries are developing adaptive regulatory approaches for AI in health research, such frameworks remain limited or absent in many LMIC contexts [40]. The African Union Data Policy Framework (2022) highlights the absence of harmonized AI governance systems across African states, resulting in fragmented and uneven regulatory responses [41]. Similarly, in East Africa, ethical oversight systems remain largely guided by general biomedical research guidelines, with limited operationalization of AI-specific review standards [42, 43]. As a result, HRECs are compelled to rely on discretionary judgment when reviewing AI protocols, which, although necessary, may introduce inconsistency and variability in ethical decision-making processes. This regulatory gap may therefore undermine the consistency, transparency, and robustness of ethical review, potentially compromising participant protection and public confidence in AI-driven research governance.

The findings reveal significant capacity limitations within HRECs in independently reviewing AI-related research protocols, particularly in areas such as algorithmic validation, bias assessment, and data science interpretation. Globally, this reflects a growing recognition that ethics committees require interdisciplinary expertise to adequately oversee AI-driven research [44]. However, many systems remain under-resourced and dependent on external technical input [42]. This reliance often results in delays and fragmented accountability structures. The African Union Data Policy Framework (2022) underscores the absence of harmonized AI governance systems across African states, resulting in fragmented and uneven regulatory responses. This lack of coordination limits the development of shared standards for data governance, algorithmic accountability, and ethical oversight, thereby constraining Africa’s collective capacity to respond coherently to emerging digital health technologies [41]. In East Africa, including Tanzania, Kenya, and Uganda, institutional review systems exhibit comparable patterns of dependence on external expertise, reflecting broader structural limitations in internal AI governance capacity [16]. For the present study, this suggests that ethical review processes for AI-related health research are likely influenced by similar capacity constraints, particularly limited in-house technical expertise and reliance on external reviewers. Such constraints may affect the consistency, depth, and timeliness of ethical decision-making. The finding, therefore, underscores the importance of assessing and strengthening local AI governance competencies within research ethics committees to support more robust, contextually grounded, and sustainable oversight of AI-driven health research.

Although resource limitations such as inadequate funding, staffing shortages, and infrastructural constraints are well-documented in LMIC ethics governance systems, this study demonstrates that AI-related research significantly intensifies these challenges. AI protocols require additional layers of review, iterative clarification, and technical consultation, thereby increasing administrative workload and extending review timelines [45]. Furthermore, the absence of digital tools for algorithm auditing and dataset validation limits committees’ ability to assess AI-specific risks in a meaningful and technically informed manner, particularly those related to bias, data quality, and model instability. This gap constrains the depth of ethical review and reduces the ability of committees to independently verify technical claims made in AI-based research protocols. Globally, the OECD (2022) and WHO (2021) emphasize that effective AI governance requires sustained investment in digital infrastructure, workforce development, and regulatory innovation [40]. Without such investment, ethics systems risk becoming increasingly misaligned with technological advancement. Digital health governance remains uneven across Africa, with many institutions facing limited access to artificial intelligence (AI) auditing tools, digital infrastructure, and technical capacity needed for effective oversight [46]. This disparity continues to constrain the implementation of robust, context-sensitive governance mechanisms for emerging digital health technologies. In East Africa, competing health system priorities further constrain investment in artificial intelligence (AI) governance infrastructure, despite the growing adoption of digital health technologies [16]. This has important implications for the present study, as it highlights the likelihood that research ethics and regulatory systems operate under resource and capacity constraints, which may limit their ability to effectively review, monitor, and govern AI-driven health research in practice. Consequently, the findings underscore the need to strengthen institutional readiness and integrate AI-specific governance capacity within existing health research oversight structures.

A critical finding of this study is the weak post-approval monitoring capacity for AI-based health research. Unlike conventional clinical studies, AI systems may evolve after approval through retraining, dataset updates, or changes in algorithmic design, resulting in dynamic ethical risks. Globally, this challenge is widely recognized, with the World Health Organization (WHO) emphasizing in its 2021 guidance that AI systems for health must be governed across their entire lifecycle, from design and development to deployment and ongoing oversight, to ensure accountability, safety, and alignment with ethical principles [47]. Similarly, the U.S. Food and Drug Administration (FDA) has advanced lifecycle-based regulatory approaches for AI-enabled medical technologies, advocating a Total Product Life Cycle (TPLC) model that integrates pre-market evaluation with continuous post-market monitoring and real-world performance assessment [48]. However, despite these global advancements, comprehensive regulatory and monitoring systems remain underdeveloped in many low- and middle-income countries (LMIC), where infrastructural, technical, and governance constraints limit effective AI oversight. Emerging evidence shows that weak digital infrastructure, limited local technical capacity, fragmented data systems, and gaps in regulatory enforcement continue to hinder the sustainable deployment and governance of AI in these settings [49]. In Africa, post-deployment surveillance systems for digital health technologies are still emerging, with limited institutional capacity to monitor algorithmic performance over time [42, 43]. This creates significant gaps in the detection of issues such as bias, drift, and unintended harm. Regionally, in East Africa, ethical review systems remain largely focused on pre-approval processes, with limited mechanisms for continuous oversight, thereby constraining long-term ethical assurance in AI-driven research [16]. This implies that critical risks, such as bias, model drift, and unintended harms, may remain undetected over time, thereby undermining patient safety, eroding public trust, and compromising the ethical integrity of AI-driven health-related research.

## Implications for HREC governance in the AI Era

The findings highlight the need for a paradigm shift in ethical governance frameworks, shifting from static, one-time approval models toward dynamic, lifecycle-based oversight systems that accommodate the evolving nature of AI technologies. Key implications include:

- Development and implementation of AI-specific ethical guidelines and updated SOPs aligned with emerging global standards.
- Integration of AI, data science, and bioinformatics expertise within HREC membership structures.
- Establishment of continuous monitoring and post-approval surveillance systems for adaptive AI technologies.
- Strengthening of digital infrastructure to support protocol tracking, data governance, and algorithmic auditing.
- Harmonization of AI ethics governance frameworks across African regulatory systems to promote consistency and shared standards.

Without such reforms, HRECs in Tanzania and other LMICs risk remaining structurally underprepared to effectively address the rapidly evolving ethical, technical, and regulatory complexities of AI-driven health research.

## Study contribution, strengths, and limitations

This study contributes empirical evidence from Tanzania to the growing global discourse on AI ethics governance in health research. It demonstrates that while systemic constraints continue to shape HREC functioning, the introduction of AI technologies adds new layers of ethical, technical, and regulatory complexity that fundamentally reshape ethical review processes. Importantly, the study shows that AI ethics oversight in LMICs is not merely an extension of existing biomedical ethics systems but requires transformative adaptation to interdisciplinary, technology-informed, and continuously responsive governance frameworks.

A key strength of this study is the inclusion of both HREC members and secretariat staff, providing complementary insights into both governance-level decision-making and operational review processes.

However, the study is limited by its purposive sampling strategy and focus on selected HRECs, which may constrain the generalizability of findings. Additionally, the study captures perceived experiences and challenges rather than direct observation of AI protocol review processes.

## Conclusion

This study explored how Health Research Ethics Committees (HRECs) in Tanzania are navigating the emerging ethical and operational complexities introduced by artificial intelligence (AI) in health research. The findings show a system anchored on strong foundational ethical governance structures, yet increasingly strained by the rapidly evolving demands of AI-driven research. While HRECs demonstrate commitment to ethical oversight through established procedures and multidisciplinary engagement, their effectiveness is constrained by limited AI-specific expertise, inadequate digital infrastructure, weak post-approval monitoring systems, and continued reliance on external technical support.

Collectively, these findings point to a widening gap between traditional ethics review systems and the dynamic, iterative nature of AI technologies. As a result, existing governance mechanisms, designed primarily for conventional biomedical research, are increasingly insufficient for addressing algorithmic opacity, evolving risk profiles, and continuous learning systems characteristic of AI applications in health research. The study therefore underscores the need for a deliberate shift toward more adaptive, lifecycle-based, and technology-responsive governance frameworks. Strengthening AI-specific capacity within HRECs, investing in digital infrastructure, institutionalizing continuous monitoring systems, and formalizing technical advisory support are essential steps to ensure robust ethical oversight. In conclusion, the future of ethical governance in health research will depend on how effectively HRECs transition from static, pre-approval-focused models to dynamic systems capable of keeping pace with technological innovation. Without such transformation, there is a risk that ethical oversight structures will lag behind the very technologies they are mandated to regulate, with implications for participant protection, public trust, and research integrity.

## Supplementary Information

Supplementary Material 1.

## Data Availability

The datasets used or analyzed during this study are available from the corresponding author upon reasonable request.

## Abbreviations

AI: Artificial Intelligence
CIOMS: Council for International Organizations of Medical Sciences
HREC: Health Research Ethics Committee
IDI: In-depth Interview
IRB: Institutional Review Board
MUHAS: Muhimbili University of Health and Allied Sciences
NatHREC: National Health Research Ethics Committee
NIMR: National Institute of Medical Research
SOPs: Standard Operating Procedures
WHO: World Health Organization
