# Supplementary material for: Exploring the operational challenges of navigating ethical oversight in the era of artificial intelligence: a qualitative study of health research ethics committees in Tanzania
Source: BMC Med Ethics. 2026 Jun 30;27:195. doi: 10.1186/s12910-026-01541-0 (PMC13579938; doi:10.1186/s12910-026-01541-0)
Supplement: Supplementary file 1 [file 12910_2026_1541_MOESM1_ESM.docx]

**Navigating Ethical Oversight in the Era of Artificial Intelligence: Perceived Factors and Operational Challenges Facing Health Research Ethics Committees in Tanzania**

**Supplemental Files**

**File 1: In-depth Interview Guide for Health Research Ethics Committee Members**

**Instructions:**

Hello, my name is **Lazaro Amon Haule**. I am a PhD student and faculty member at the Muhimbili University of Health and Allied Sciences (MUHAS). I am conducting a study that explores how Health Research Ethics Committees navigate ethical review and operational responsibilities in the context of artificial intelligence in health research. This interview aims to understand your experiences and perspectives on factors influencing ethical review processes, as well as the operational challenges faced by HRECs in the era of AI.

Your participation is voluntary. You may choose not to answer any question or to stop the interview at any time. All information shared will be kept confidential, and no identifying details will be included in any reports or publications. With your permission, I would like to audio-record this interview to ensure accuracy. If you prefer not to be recorded, I will take written notes instead. There are no right or wrong answers; please feel free to share your views openly.

Thank you for your time and participation.

**A. Administrative and Governance Structures**

**I. Policies, SOPs, and Guidelines**

- Stem question: What policies, SOPs, or guidelines guide your review of research protocols?

**Probes:**

- Are these SOPs specific to AI or emerging technologies?
- How do you handle situations not covered by SOPs?
- Can you give an example of a recent protocol where guidelines were unclear?

**II. Committee Autonomy and Governance**

- Stem question: To what extent can your committee make independent decisions?

**Probes:**

- Have higher authorities ever influenced your decisions?
- How does autonomy affect your ability to review complex or AI-driven studies?
- What happens when committee decisions are overridden or delayed?

**III. Committee Composition and Expertise**

- Stem question: How would you describe the diversity and expertise of your committee members?

**Probes:**

- Are all relevant disciplines represented (medical, legal, social sciences, IT, AI)?
- How does turnover affect committee functioning?
- Do you feel any expertise is missing for reviewing AI-related studies?

**IV. Documentation and Tracking**

- Stem question: How does your committee track submissions, approvals, and ongoing studies?

**Probes:**

- Have you experienced delays or errors in tracking protocols?
- Are AI-related protocols more difficult to track? How?
- What improvements would help in documentation and follow-up?

**B. Operational and Resource Constraints**

**I. Financial and Material Resources**

- Stem question: Are financial resources sufficient for committee activities, including meetings and stipends?

**Probes:**

- How do funding limitations affect review timelines or quality?
- Does the reliance on investigator fees create challenges?

**II. Workload and Human Resources**

- Stem question: How would you describe the workload of committee members?

**Probes:**

- How do competing professional responsibilities affect review quality?
- Are delays or backlogs common? Why?
- How do AI protocols affect workload differently from conventional studies?

**C. Capacity, Training, and Expertise Gaps**

**I. AI-Specific Skills**

- Stem question: Have you received training relevant to AI or emerging technologies?

**Probes:**

- What specific areas of AI research are most challenging to review?
- How would additional training improve your review process?

**II. Reliance on External Experts**

- Stem question: How often do you consult external advisors?

**Probes:**

- What challenges arise when coordinating external reviewers?
- Do delays occur due to external consultations?

**D. Monitoring, Compliance, and Post-Approval Oversight**

**I. Post-Approval Monitoring**

- Stem question: How does your committee ensure compliance of approved studies?

**Probes:**

- Are there systematic follow-up procedures?
- How are AI-specific risks monitored post-approval?

**II. Ethical Risk Assessment**

- Stem question: How confident are you in assessing ethical risks of complex studies?

**Probes:**

- What makes AI protocols particularly difficult to evaluate?
- What tools or resources would help improve risk assessment?

**Closing:**

1. What do you consider the main factors limiting effective ethical review?

**Probe:** Can you provide examples from recent experiences?

2. What recommendations would you make to strengthen HREC functioning, especially for AI research?

**File 2: In-depth Interview Guide for HREC Secretariats - Key Informants**

**Instructions:**

This interview seeks to understand your experiences as an HREC Secretariat member, particularly regarding administrative, coordination, and operational aspects of ethical review in the context of AI-related health research. Your participation is entirely voluntary. You may decline to answer any question or withdraw from the interview at any point without any consequences.

All information you provide will be treated with strict confidentiality, and no identifying information will be disclosed. With your permission, the interview will be audio-recorded to ensure accurate documentation. If you prefer not to be recorded, notes will be taken instead. Please feel free to share your experiences openly; there are no right or wrong responses.

Thank you very much for your time and contribution to this study.

**A. Administrative and Governance Structures**

**I. SOPs and Guidelines**

- Stem question: What SOPs or policies guide your administrative support for protocol review?

**Probes:**

- Are they adequate for AI research?
- How do you manage cases not clearly covered by SOPs?
- Can you share an example of challenges with AI protocols?

**II. Committee Autonomy and Governance**

- Stem question: To what extent can the HREC operate independently?

**Probes:**

- How do higher authorities or institutional structures affect your administrative work?
- Have you encountered delays caused by required approvals from higher authorities?

**III. Committee Composition and Expertise**

- Stem question: How does the committee’s expertise and diversity affect your role?

**Probes:**

- How do you coordinate with external technical advisors?
- Are there expertise gaps that affect your work?

**IV. Documentation and Tracking Systems**

- Stem question: How are protocols tracked and documented?

**Probes:**

- How difficult is it to track AI-driven protocols?
- Have you experienced errors or lost data?
- What improvements could help your workflow?

**B. Operational and Resource Constraints**

**I. Financial and Material Resources**

- Stem question: Are resources adequate for HREC administration, meetings, and oversight?

**Probes:**

- How do resource limitations affect protocol review?
- Does reliance on submission fees affect objectivity or efficiency?

**II. Workload and Staffing**

- Stem question: How sufficient is secretariat staff support?

**Probes:**

- Are workloads manageable for AI-related protocols?
- How do delays or backlogs occur?

**3. IT and Analytical Tools**

- Stem question: Do you have access to IT systems or analytical tools for reviewing or monitoring complex studies?

**Probes:**

- Are tools adequate for AI protocols?
- What improvements are needed?

**C. Capacity, Training, and Expertise**

**I. AI-Specific Knowledge**

- Stem question: Have you received training on AI research ethics?

**Probes:**

- Which areas are most challenging for your administrative role?
- What additional training or support would help?

**II. Dependence on External Expertise**

- Stem question: How frequently do you coordinate with external technical advisors?

**Probes:**

- What logistical challenges arise?
- How does this affect review timelines?

**D. Monitoring, Compliance, and Post-Approval Oversight**

**I. Post-Approval Monitoring**

- How are ongoing studies tracked for compliance?

**Probes:**

- How do resource limitations affect monitoring?
- Are AI protocols particularly difficult to track?

**II. Ethical Risk Assessment**

- Stem question: How confident are you in identifying risks related to complex or AI-driven studies?

**Probes:**

- Which ethical risks are most difficult to assess?
- What support would help improve oversight?

**Closing:**

1. What are the main operational barriers to effective HREC performance?

**Probe:** Can you give recent examples?

2. What strategies would you recommend to strengthen HREC functionality, particularly for AI research?
